# Prediction of Protein–Protein Interactions with Clustered Amino Acids and Weighted Sparse Representation

**DOI:** 10.3390/ijms160510855

**Published:** 2015-05-13

**Authors:** Qiaoying Huang, Zhuhong You, Xiaofeng Zhang, Yong Zhou

**Affiliations:** 1 Shenzhen Graduate School, Harbin Institute of Technology, HIT Campus of University Town of Shenzhen, Shenzhen 518055, China; E-Mails: charwinghuang@gmail.com (Q.H.); zhangxiaofeng@gmail.com (X.Z.); 2 School of Computer Science and Technology, China University of Mining and Technology, Xuzhou 221116, China; E-Mail: yzhou@cumt.edu.cn

**Keywords:** reduced amino acid alphabet, weighted sparse representation-based classification, protein–protein interactions

## Abstract

With the completion of the Human Genome Project, bioscience has entered into the era of the genome and proteome. Therefore, protein–protein interactions (PPIs) research is becoming more and more important. Life activities and the protein–protein interactions are inseparable, such as DNA synthesis, gene transcription activation, protein translation, *etc*. Though many methods based on biological experiments and machine learning have been proposed, they all spent a long time to learn and obtained an imprecise accuracy. How to efficiently and accurately predict PPIs is still a big challenge. To take up such a challenge, we developed a new predictor by incorporating the reduced amino acid alphabet (RAAA) information into the general form of pseudo-amino acid composition (PseAAC) and with the weighted sparse representation-based classification (WSRC). The remarkable advantages of introducing the reduced amino acid alphabet is being able to avoid the notorious dimensionality disaster or overfitting problem in statistical prediction. Additionally, experiments have proven that our method achieved good performance in both a low- and high-dimensional feature space. Among all of the experiments performed on the PPIs data of *Saccharomyces cerevisiae*, the best one achieved 90.91% accuracy, 94.17% sensitivity, 87.22% precision and a 83.43% Matthews correlation coefficient (MCC) value. In order to evaluate the prediction ability of our method, extensive experiments are performed to compare with the state-of-the-art technique, support vector machine (SVM). The achieved results show that the proposed approach is very promising for predicting PPIs, and it can be a helpful supplement for PPIs prediction.

## 1. Introduction

Protein–protein interactions (PPIs) stand for the intentional physical contacts built between multiple proteins for proper biological activities [1]. Generally, PPIs play vital roles in diverse essential molecular processes, including signal transduction [2], cell metabolism and muscle contraction [3]. With the increasing research attention on PPIs, a number of approaches have been proposed to investigate how they interact [4]. In the existing literature, the most widely-adopted experimental technologies are yeast two-hybrid (Y2H) [5] and tandem affinity purification (TAP) [6]. However, the computational process of both of the aforementioned biological techniques is time consuming. In addition, the accuracy of these approaches is still not satisfying. To resolve these two issues simultaneously, efficient computational approaches are required for the effective analysis of PPIs [7,8,9,10,11].

Thereafter, a number of computational approaches have been proposed to speed up the prediction process of PPIs [12]. Nevertheless, with the scale of protein sequences getting larger and larger, most of the existing computational approaches become invalid due to the following reasons. These methods are generally proposed to cope with various data types, such as protein domain, genomic information and protein structure information, and the prior information of protein pairs is needed to properly predict PPIs. However, the data complexity also increases when the data scale gets large; such protein pairs are hard to directly obtain and, thus, invalidate these computational approaches. Therefore, the protein sequence-based approaches are preferred, as they directly derive the necessary information from the amino acid sequence. Recently, Hosur *et al.* [13] proposed a threading-based approach to predict PPIs directly based on protein sequences. Additionally, Guilherme Valente *et al.* [14] named their approach Universal *In Silico* Predictor of Protein–Protein Interactions (UNISPPI), which classified PPIs based on the original protein sequence information with a satisfying accuracy.

As is known, the support vector machine (SVM)-based approach is one of the most effective classification methods, which has been successfully applied to protein remote homology detection [15,16,17], DNA binding protein identification [18], *etc.* In the study of Shen *et al.* [19], 20 amino acids were classified into seven categories according to the side chain of the dipole, as well as their volume, and then, the features of protein pairs were extracted using the conjoint triad method on the basis of the previous classification results on their amino acids. When evaluated on a human PPIs dataset, this approach achieves a higher prediction accuracy. However, the conjoint triad method ignores both the effect of neighbors on PPIs and the interactions occurring between discontinuous segments of amino acid sequences. Guo *et al.* [20] exploited automatically extracting the covariance features of protein sequences and predicted PPIs via support vector machine on a Saccharomyces cerevisiae dataset. Although these approaches could achieve higher prediction accuracy, their prediction performance greatly relies on the model selection procedure, and therefore, a stable prediction result cannot be guaranteed.

There are two key factors affecting the predicting performance of PPIs, *i.e.*, feature extraction and sample classifier. Obviously, feature extraction is vital for all kinds of classifiers. In genome analysis, a well-selected feature generally helps to reveal hidden relationships between proteins and their biological activities. There already exists a wide range of feature selection approaches to extract features out of amino acid compositions, but few approaches consider the effect of amino sequence order. Chou *et al.* [21] proposed to use pseudo-amino acid composition for further analysis. Based on their approach, they identified 20 factors only reflecting the influence of amino acid composition, whereas the rest of the factors are influenced by the sequence order. This approach is then widely adopted by many protein attributes-based prediction approaches, such as predicting amino acid gamma-aminobutyric-acid (GABA(A)) receptor proteins [22], predicting protein folding rates [23], identifying cyclin proteins [2], predicting supersecondary structure [24] and predicting a protein’s subcellular location [25,26]. More similar works could be found in the review paper of Gonzalez-Diaz *et al.* [27]. Recently, two open-access tools were released to generate various modes of Chou’s pseudo-amino acid composition [28,29,30]. One of the most useful, pseudo-amino acid composition (PseAAC) modes, is the so-called *n*-peptide composition; however, the dimension of features exponentially increases when we increase *n*. To avoid the dimensionality explosion problem, we, in this paper, attempt to integrate the approach of the reduced amino acid alphabet [31,32,33] with the general form of PseAAC to constrain the increase of feature dimensions.

For the sample classifier, the most popular choice is SVM; however, it needs a careful parameter tuning process in order to achieve better performance, and this tuning process requires extra efforts [34,35]. To cope with this issue, sparse representation classification (SRC) [36] can be adopted, which was originally proposed for face recognition. As indicated in [37], the original SRC ignores the relationship between features in low-dimensional space, which is not desired. Therefore, we determine to adopt a weighted sparse representation-based classification approach, which does not require a longer process in parameter tuning, but a comparable good classification accuracy.

The rest of this paper is organized as follows: (1) we first generate a benchmark dataset for the validation of our proposed method; (2) we then introduce an efficient feature extraction approach that can discover the intrinsic correlation between proteins; (3) we adopt a powerful classification algorithm to predict PPIs; (4) we execute the cross-validation tests to evaluate the prediction accuracy; and (5) we further evaluate our method on other dataset and compare it with other feature extraction methods and classifier.

## 2. Results and Discussion

### 2.1. Evaluation Criteria

To evaluate the performance of our approach, the following criteria are chosen in the experiments, which are the accuracy (ACC), sensitivity (SN), precision (PE) and Matthews correlation coefficient (MCC), written as:

(1) $$\text{ACC}=\;\frac{\text{TP}+\text{TN}}{\text{TP}+\text{FP}+\text{TN}+\text{FN}}$$

(2) $$\text{SN}=\;\frac{\text{TP}}{\text{TP }+\text{ FN}}$$

(3) $$\text{PE}=\;\frac{\text{TP}}{\text{TP }+\text{ FP}}$$

(4) $$\text{MCC}=\;\frac{\text{TP }\times \text{ TN }-\text{FP }\times \text{ FN }}{\surd \;\left(\text{TP}+\text{FN}\right)\;\times \left(\text{TN }+\text{ FP}\right)\;\times \;\left(\text{TP }+\text{ FP}\right)\;\times \;\left(\text{TN }+\text{ FN}\right)}$$

where TP, TN, FP and FN denote true positive, true negative, false positive and false negative, respectively. In addition, the receiver operating characteristic (ROC) curve is also adopted to evaluate the prediction performance. The ROC curve plots the true positive rate (TPR) *versus* the false positive rate (FPR) with the threshold varying. The area under the ROC curve is called the area under the ROC curve (AUC), which falls into (0,1). The larger the AUC, the better prediction performance we can achieve.

### 2.2. Assessment of Prediction Ability

The Gaussian kernel width σ and the tolerance threshold ε are two parameters that need to be tuned in the experiments. After a careful parameter tuning, we set σ = 50 and ε = 0*.*05 in the rest of the experiments.

**Figure 1:**
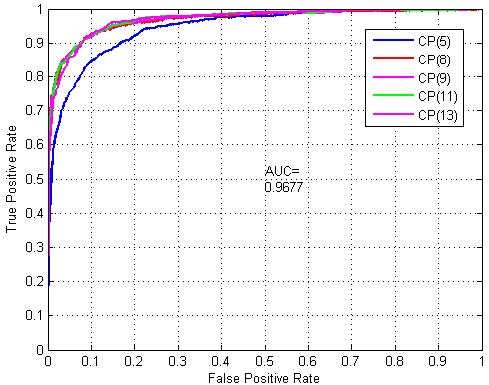
Receiver Operating Characteristic (ROC) curve.

In the following experiments, we used five-fold cross-validation to perform the experiments. Several models were constructed and evaluated on five different cluster profiles (shown in Table 1) for the dipeptide case (shown in Table 2, *n* = 1, 2, 3). The prediction results are reported in Table 3. It is obvious that when we increase *n*, the prediction performance of our approach improves and achieves the best performance when *n* = 3. From Table 3, we can observe that the prediction result is the best for CP(8) with dimension = 512, and its accuracy is 90.91%, sensitivity 94.17%, precision 87.22% and MCC value 84.43%. In general, the PPI prediction accuracy of our approach is higher than 70%, and most of the 15 groups are better than 80%. Moreover, we plotted the ROC curve and AUC values of CP(8) with dimension = 512, as shown in Figure 1, and its AUC value is 0.97307, which is close to one.

**Table 1:** Scheme for reduced amino acid alphabet based on protein blocks method.

Cluster Profiles	Protein Blocks Method
CP(13)	G-IV-FYW-A-L-M-E-QRK-P-ND-HS-T-C
CP(11)	G-IV-FYW-A-LM-EQRK-P-ND-HS-T-C
CP(9)	G-IV-FYW-ALM-EQRK-P-ND-HS-TC
CP(8)	G-IV-FYW-ALM-EQRK-P-ND-HSTC
CP(5)	G-IVFYW-ALMEQRK-P-NDHSTC

**Table 2:** The dimension of feature vectors of *n*-peptide composition with different cluster profiles.

*n*-Peptide	CP(13)	CP(11)	CP(9)	CP(8)	CP(5)
*n* = 1	13	11	9	8	5
*n* = 2	169	121	81	64	25
*n* = 3	2197	1331	729	512	125

**Table 3:** Five-fold cross-validation results of protein–protein interactions (PPIs) prediction. ACC, accuracy; SN, sensitivity; PE, precision; MCC, Matthews correlation coefficient. (The numbers in bold is the best result.).

Criteria	CP(5)			CP(8)			CP(9)			CP(11)			CP(13)		
Dimension	5	25	125	8	64	512	9	81	729	11	121	1331	13	169	2197
ACC (%)	71.16	82.85	87.43	77.90	87.74	**90.91**	79.63	88.19	90.86	81.47	89.05	90.77	82.99	89.46	90.26
SN (%)	71.26	84.21	89.88	78.02	90.02	**94.17**	79.92	90.72	94.15	81.78	91.52	**94.17**	83.75	92.23	93.32
PE (%)	70.94	80.86	84.38	77.70	84.90	**87.22**	79.14	85.10	87.13	80.99	86.08	86.91	81.88	86.19	86.71
MCC (%)	58.96	71.55	77.98	65.57	78.45	**83.43**	67.56	79.14	83.34	69.81	80.47	83.19	71.76	81.11	82.37

### 2.3. Robustness Performance Evaluation

In order to evaluate the robustness of our approach, we further test our algorithm on an H. pylori dataset. This dataset includes 1365 interaction protein pairs, as well as some noise. Those proteins with the number of amino acids <50 will be removed from the dataset. The prediction classifier is built with 1365 × 2 = 2730 protein pairs and keeps the same parameter settings. The prediction results are reported in Table 4. The accuracy of our approach is the highest (83.04%) on CP(13) with dimension = 169. From this table, the accuracy does not improve as *n* increases, for example CP(11) with dimension = 121 and 1331 and CP(13) with dimension = 169 and 2197. The reason is the existence of “overfitting” or “high dimensionality disaster” problems. From this observation, we can conclude that by properly choosing feature dimensions, we can save computational cost, as well as improve the prediction accuracy.

**Table 4:** Prediction results on the H. pylori dataset. (The numbers in bold is the best result.).

Criteria	CP(5)			CP(8)			CP(9)			CP(11)			CP(13)		
Dimension	5	25	125	8	64	512	9	81	729	11	121	1331	13	169	2197
ACC (%)	66.63	76.34	81.43	72.89	81.68	81.90	73.19	81.79	80.81	74.43	82.60	78.50	76.19	**83.04**	77.29
SN (%)	64.76	72.45	77.67	70.67	77.90	77.56	70.44	78.39	76.27	71.29	78.77	73.41	72.61	**79.20**	71.69
PE (%)	72.65	85.12	88.47	78.18	88.51	89.94	79.89	88.03	89.49	81.67	89.41	89.72	84.03	89.75	**90.37**
MCC (%)	55.14	63.29	69.49	60.28	69.75	70.02	60.40	69.96	68.53	61.56	71.00	65.38	63.29	**71.60**	63.73

### 2.4. Comparison with Other Methods

To further demonstrate the effectiveness of our approach, several related state-of-the-art approaches are performed in this experiment for performance comparison. We first implement several weighted sparse representation-based classification (WSRC) classifiers using different feature extraction approaches, and we also perform the SVM classifier with the pseudo-amino acid composition and reduced amino acid alphabet feature.

For the feature extraction method, we chose the most popular feature extraction methods in PPIs prediction, which are auto covariance, Moran autocorrelation, Geary autocorrelation, conjoint triad and pseudo-amino acid composition. The experiments are also performed using five-fold cross-validation. As shown in Table 5, the WSRC classifier with the pseudo-amino acid composition and reduced amino acid alphabet feature can achieve better results than the other methods in terms of accuracy, sensitivity, precision and MCC values.

**Table 5:** Comparisons of different feature extraction methods.

Criteria	Auto Covariance	Conjoint Triad	Moran Autocorrelation	Geary Autocorrelation	Pseudo-Amino Acid Composition	Our Method
ACC (%)	88.89	89.57	88.90	85.06	86.45	90.91
SN (%)	92.07	92.91	92.12	88.20	88.16	94.17
PE (%)	85.12	85.68	85.08	80.97	84.23	87.22
MCC (%)	80.19	81.26	80.21	74.51	76.56	84.43

The SVM classifier with the same feature set is performed and is compared with the WSRC classifier. The parameters of the SVM classifier need to be tuned, which are soft margin parameter *C* and Gaussian kernel parameter *g*. In this experiment, we set *C* = 16 and *g* = 16. As shown in Table 6, the SVM classifier can achieve the best results for CP(13) with dimension = 2197, and its accuracy, sensitivity, precision and MCC are 92.04%, 90.37%, 93.51% and 85.35%, respectively, which are a little higher than the best results shown in Table 5. However, the WSRC classifier can achieve higher accuracies in most of the tested 15 groups. Especially, the WSRC significantly outperforms the SVM classifier in the low-dimensional data space. In the high-dimensional data space, the SVM performs very well, but with a higher computational cost. In fact, the performance of SVM seriously relies on its parameters, and the performance can vary greatly if the parameters are not properly tuned, whereas the performance of the WSRC classifier is quite stable and varies slightly when the parameters change. To emphasize once more, the proposed WSRC can achieve comparable classification performance with most of the experimental settings and is especially good in the low-dimensional data space. Alternatively, by performing the PPIs prediction in a low-dimensional data space, we can largely save the computational cost, as well as preserve the relationships between non-continuous protein pairs. However, the SVM-based classifier can achieve better performance only in a high-dimensional data space, which indicates that a much higher time complexity is required to achieve the best model performance. In practice, it is desired that the PPIs classifier can achieve better classification performance with a much lower cost. Apparently, the proposed WSRC is superior to the SVM, as well as the WSRC, with other feature extraction methods.

**Table 6:** Comparisons of the weighted sparse representation-based classification (WSRC) and support vector machine (SVM). (The numbers in bold is the best result.).

Criteria	CP(5)			CP(8)			CP(9)			CP(11)			CP(13)		
Dimension	5	25	125	8	64	512	9	81	729	11	121	1331	13	169	2197
ACC (%)	67.27	74.80	81.44	72.05	82.81	88.17	72.86	83.32	89.23	74.58	84.89	90.81	75.66	86.27	**92.04**
SN (%)	71.04	75.56	80.49	75.20	82.64	86.35	75.62	83.18	87.48	77.20	84.45	88.85	77.60	85.46	**90.37**
PE (%)	66.06	74.44	82.06	70.76	82.95	89.61	71.69	83.43	90.66	73.36	85.22	92.48	74.71	86.88	**93.51**
MCC (%)	55.84	62.29	69.78	59.64	71.53	79.12	60.39	72.21	80.76	62.03	74.34	83.30	63.14	76.31	**85.35**

We further compared our best result with previous reported papers, such as Guo *et al.* [20], Zhou *et al.* [38] and Yang *et al.* [39]. Additionally, we also implemented sparse representation-based classification (SRC) and SVM under the same setting as WSRC, which used CP(8) with dimension = 512. The results are shown in Table 7. As we see, WSRC gains the best performance in accuracy, sensitive and MCC with values of 90.91%, 94.17% and 83.43%, respectively. Though WSRC cannot beat Yang’s work in precision, it still reveals the potential of our method in predicting PPIs.

**Table 7:** Comparisons of WSRC with other classifiers. (The numbers in bold is the best result.).

Criteria	WSRC	SRC	SVM	Guo’s Work	Zhou’s Work	Yang’s Work
ACC (%)	**90.91**	87.46	88.17	89.33	88.56	86.15
SN (%)	**94.17**	89.93	86.55	89.93	87.37	81.03
PE (%)	87.22	84.39	89.61	88.87	89.50	**90.24**
MCC (%)	**83.43**	78.02	79.12	N/A	77.15	N/A

## 3. Experimental Section

### 3.1. Dataset

The PPI dataset was manually extracted from the public *S. cerevisiae* subset of interacting proteins (DIP) database [20]. First, to reduce the size of the dataset, we removed the protein pairs whose length is less than 50 residues, and we also removed those that have more than 40% sequence identities. A positive sub-dataset (protein pairs with interaction) is generated consisting of 5594 protein pairs. To generate the negative sub-dataset (protein pairs without interaction), we also assume that proteins from different subcellular positions will not interact with each other. Accordingly, the negative dataset with 5594 protein pairs is generated, and the whole evaluation dataset is acquired, containing 5594 positive protein pairs and 5594 negative protein pairs.

### 3.2. Pseudo-Amino Acid Composition and Reduced Amino Acid Alphabet

Given a protein sequence, how to properly represent the protein sequence is one of the key issues in protein–protein interaction prediction. Conventionally, a protein can be represented by its entire amino acid sequence as it contains complete information. For a protein amino acid sequence with *L* residues, its representation can be written as:

*P* = *R*_1_···*R*_L_ (5)

where *R*_1_ denote the first residue of protein *P* and there are a total of *L* residues in *P*. As proposed in BLAST [40], the similarity between a series of amino acid sequences is calculated as the basis for the later prediction. However, the prediction fails when the query protein does not have significant homologies to the known proteins. Alternatively, discrete models are proposed, which do not rely on the order of amino acid sequences for the similarity calculation. One of the most commonly-adopted discrete model is to use the amino acid composition (AAC) to represent proteins, which is formulated as:

*P* = [*f*_1_···*f*_20_]*^T^* (6)

where *f_u_* (*i* = 1···20) is a normalized occurrence frequency of the 20 amino acids in *P*, and *T* is the transpose operator. As a result, once the protein sequence information is known, the amino acid composition of a protein can be easily calculated. By formulating with Equation (6), the effect of sequence order is not considered, and thus, we propose to a new approach, which is based on pseudo-amino acid composition (PseAAC). The new approach can now make use of the characteristics of amino acid composition, as well as the order of the amino acid sequence.

Among all of the PseAAC modes, the simplest one is the so-called *n*-peptide composition. When *n* = 1, 2, 3, this model degenerates to the AAC model, dipeptide composition [41,42,43] and tripeptide composition [44], respectively. In this way, the sort of sequence order information can be preserved. However, when *n* ≥ 2, the number of components increases rapidly. For example, when *n* = 2, there are around 20^2^ = 400 features generated for the prediction, whereas we should make the prediction using over 20^3^ = 8000 features when *n* = 3. The exponential increase of features not only leads to an unacceptable model training process, but also causes a very expensive biological experiment in terms of both the experimental materials and the experiment process. In addition, this high-dimensional feature set will also generate a series of other issues, such as: (i) the over-fitting problem, which will cause poor model generalization; and (ii) the sparse representation problem, which will cause serious biased results and a poor ability to interpret missing data.

To avoid this well-known issue, we propose to use the reduced amino acid alphabet (RAAA) approach and clustering 20 native amino acids into a smaller number of groups with each group as a representative residue. Compared with the traditional AAC approach, RAAA not only considers the protein composition, but also preserves the sequence order information. By assuming that different amino acids might be responsible for the same biological activity, it can group similar amino acids into the same group. With the clustering process, this approach can greatly reduce the dimension of features used. Later, De Brevern *et al.* proposed a structural alphabet, called protein blocks (PBs) [45,46], which has been widely applied in computational proteomics [47,48,49]. To further improve the efficiency, a novel RAAA was proposed by Etchebest *et al.* based on PBs [50], where the 20 native amino acids are grouped into five different cluster profiles, that is CP(13), CP(11), CP(9), CP(8) and CP(5), as shown in Table 1.

Accordingly, the proteins are now encoded with RAAA by a discrete feature vector *P* written as:

*P* = [*f*_1_, *f*_2_···*f_i_*···]*^T^* (7)

where *T* is the transpose operator and *f_i_*is the occurrence frequency of the *i*^th^ *n*-peptide RAAA. For instance, using CP(13) and *n* = 1, *G* is grouped into the first group; *I*/*V* fall into the second group, and so on. As *I* and *V* are in the same group, their occurrence frequencies are counted in the second group. Clustered into 13 groups, the dimension of the feature vector of CP(13) is only 13, which is much smaller than that of the traditional AAC features, *i.e.*, 20. When *n* = 2, *GG* is in the first group, *GI*/*GV*/*IG*/*VG* are in the second group, and similarly, we can get the rest of the groups. To summarize, the elements in these 13 groups can permute to form 169 new groups. Table 1 shows the dimension of feature vectors of Equation (7) with different cluster profiles and different *n*, and the corresponding dimensions are reported in Table 2. Apparently, we can observe that the dimension of the feature vector generating the adopted approach is much smaller than those generated by the traditional dipeptide composition and tripeptide composition. By this comparison, we determine the approach to represent features of amino acids, and we then introduce one effective algorithm for PPIs prediction.

### 3.3. Weighted Sparse Representation Based Classification

Sparse representation classification (SRC) assumes that there is a training sample matrix *X* ∈ **R***^d^*^×*n*^ with *n* samples and d-dimensional feature vectors. Let *c_l_* denote the *l^th^* sample of *X* and all the samples are divided into *K* object classes. Assuming that there are *n_i_* samples belong to the *i^th^* class, *X_i_* = [*c_i_*_1_···*c_in__i_*] is the whole data set which can be rewritten as *X* = [*X*_1_···*X_K_*]. Suppose there is a new testing sample *y* ∈ **R***^d^* belongs to the *i^th^* class, the sparse representation is to find a column vector α = [α*_i_*_1_···α*_in__i_*] such that:

*y* = α*_i_*_1_*c_i_*_1_ + ··· + α*_in__i_c_in__i_* (8)

Suppose a linear representation coefficient vector α_0_∈ R*^n^*, *y* can be written in terms of all training samples as:

*y* = *X*α_0_ (9)

According to the sparse representation approach, in α_0_, only the entries corresponding to the same class as *y* have a nonzero value. Then, we have:

α_0_ = [0,···,0, α*_i_*_1_,α*_i_*_2_,···,α*_ini_*,0,···,0] (10)

The SRC aims to solve the following *l*_0_ minimization problem:

αˆ_0_ = *argmin* ‖α‖_0_ *s.t.y* = *X*α (11)

Theoretically, it is an NP hard problem to solve Equation (11) [51]. To solve this problem, we replace this equation with its convex surrogates, as mentioned by Candes [52], for which the *l*_1_ minimum solution approximates to that of *l*_0_ solutions. Therefore, Equation (11) is rewritten as:

αˆ_1_ = *argmin* ‖α‖_1_ *s.t.y* = *X*α (12)

To avoid the occlusion problem, the *l*_1_ norm minimization is further extended to the following stable *l*_1_ norm minimization problem as:

αˆ_1_ = *argmin* ‖α‖_1_ *s.t.* ‖y − *X*α‖ ≤ ε (13)

where ε *>* 0 is a pre-defined threshold. This minimization can be resolved using standard linear programming methods [53]. After obtaining the sparsest solution αˆ_1_, SRC uses the following classification criterion denoted as:

*g_k_*(*y*) = ‖*y* − *X*αˆ_1*k*_‖, *k* = 1···*K* (14)

where *y* belongs to class *k* when the minimum solution Equation (14) is acquired. As mentioned before, only when *y* belongs to class *k* do those entries of αˆ_1_ associated with class *k* have a nonzero value. As *g_k_* represents the residual, we want to assign *y* to the smallest residual.

However, as pointed out by [37], the SRC can only achieve better performance when the data are represented by a high-dimensional feature space, which contradicts our method representing amino acids in a low-dimensional feature space. The weighted sparse representation-based classification (WSRC) approach proposed in [37] can solve this problem well, and it also can guarantee a better performance in the low-dimensional data space. To adopt this approach, we need further to study how to properly choose α, as it determines the residuals. The goal of WSRC is to find a proper way to evaluate the relationship between training and testing samples. The natural measurement metric is to compare the distance between training and test samples. The algorithm has two main steps, and the first step is to calculate the distance between existing training samples and given test data by which we can calculate a new α. The second step is to run the traditional SRC algorithm with the new α.

In this paper, we choose the Gaussian kernel to compute the data distance, as the Gaussian kernel distance can capture the nonlinear relationship in the dataset. With this Gaussian kernel distance, we can then calculate the similarity between the training data and testing data. The data samples are represented as *x*,*y* ∈ **R***^d^*, where *x*,*y* is the training and testing sample, respectively. Additionally, their Gaussian kernel distance can be written as:

*d_g_*(*x*,*y*) = *exp*(−‖*x* − *y*‖^2^*/*2σ^2^) (15)

where σ is the scale parameter of the Gaussian kernel. Compared with the traditional distance measurement [54], such as the Euclidean distance, the Gaussian kernel distance can preserve the neighborhood relationship well in the nonlinear data space. It is also the first attempt to adopt Gaussian kernel distance for calculating the weights for SRC. As the Gaussian kernel distance is between 0 and 1, we can directly use this distance as the weight of the training samples. Therefore, for a training sample *x_i_*∈ **R***^d^*, its weight can be written as *d_g_*(*x_i_*,*y*). By calculating the weight of each training sample, a new weighted training dataset can be generated, which is denoted as *X*' = [*X*'_1_,··· *X*'*_C_*]. Additionally, for the *k*^th^ class, *X*'*_k_* = [*w_k_*_1_*X_k_*_1_,···,*w_kn__k_X_kn__k_*], where *n_k_* is the number of training samples in the *k*^th^ class. Consequently, the new WSRC classifier can be learned, and the corresponding algorithm is illustrated in Algorithm 1.

Algorithm 1: The WSRC Algorithm

1: Normalize the columns of *X* to have unit *l*_2_-norm.

2: Give a new test sample *y*; calculate the distance between each training sample *x* via Gaussian kernel *d_g_*(*x*,*y*) = *exp*(−‖*x* − *y*‖^2^*/*2σ^2^). Use the results to determine the weight and to generate the new training set:

*X*' = [*X*'_1_,··· *X*'*_C_*] (16)

3: Find a column vector α satisfying the following formulation:

αˆ_1_ = *argmin* ‖α‖_1_ *s.t.* ‖*y* − *X*α‖ ≤ ε (17)

4: Compute the residuals:

*g_k_*(*y*) = ‖*y* − *X*αˆ_1*k*_‖, *k* = 1···*K* (18)

where αˆ_1*k*_ is the representation coefficient vector associated with class *k.*

5: Output the class label of *y* as *k* = identity (argmin *g_k_*(*y*))

Compared with traditional SRC, WSRC can utilize the similarity between training and testing samples well with a linear sparse feature representation. It can significantly improve both the robustness and the accuracy of the PPIs prediction. For the parameter learning, we only need to carefully tune σ, the Gaussian kernel width and the threshold ε. In the following section, we will discuss how the experiments are performed, as well as the related discussion.

## 4. Conclusions

In this paper, we proposed a new PPIs prediction method, which makes the best use of the information of protein sequence order. The proposed prediction model was built based on the WSRC classifier and integrated with both the pseudo-amino acid composition and the reduced amino acid alphabet feature. Unlike the *n*-peptide method, we can acquire a set of features combining the PseAAC and RAAA feature with different feature dimensions. This novel feature extraction method can effectively reduce the data dimension and reduce the computational cost. In the experiments, we can see that the performance of WSRC with the features extracted by our method is superior to the state-of-the-art SVM in terms of accuracy, sensitivity, precision and MCC value. This is the first attempt to adopt WSRC to predict PPIs. The promising experimental results demonstrate that the WSRC approach can not only achieve better performance with the PseAAC feature combined with RAAA, but also with other features. This shows the practical significance of our method in predicting PPIs.
